# Know your scientist: KYC as biosecurity infrastructure

**DOI:** 10.3389/fmicb.2026.1814993

**Published:** 2026-04-29

**Authors:** Jonathan Feldman, Tal Feldman, Annie I. Anton

**Affiliations:** 1 College of Computing, Georgia Institute of Technology, Atlanta, GA, United States; 2 Yale Law School, New Haven, CT, United States

**Keywords:** access control, AI governance, biological design tools, biosecurity, dual-use risk, KYC

## Abstract

Biological AI tools for protein design and structure prediction are advancing rapidly, creating dual-use risks that existing safeguards cannot adequately address. Current model-level restrictions, including keyword filtering, output screening, and content-based access denials, are fundamentally ill-suited to biology, where reliable function prediction remains beyond reach and novel threats evade detection by design. Because the full spectrum of risks cannot be managed by any single actor, effective oversight requires shared responsibility between research institutions and model hosts. Hence, we propose a three-tier Know Your Customer (KYC) framework, inspired by anti-money laundering (AML) practices in the financial sector, that augments existing approaches, supplementing content inspection with complementary layers of user verification and monitoring. Tier I leverages research institutions as trust anchors to vouch for affiliated researchers and assume responsibility for vetting. Tier II applies output screening through sequence homology searches and functional annotation. Tier III monitors behavioral patterns to detect anomalies inconsistent with declared research purposes. This layered approach preserves access for legitimate researchers while raising the cost of misuse through institutional accountability and traceability. The framework can be implemented immediately using existing institutional infrastructure, requiring no new legislation or regulatory mandates.

## 1 Introduction

Advances in artificial intelligence are rapidly expanding the capabilities of biological research tools. Systems for structure prediction, sequence modeling, and generative protein design now support tasks that were recently limited to specialized laboratories (Ruffolo and Madani, 2024; Chen et al., 2023; Xiao et al., 2025; Meier et al., 2021; Hie et al., 2022; Huot et al., 2025; Feldman and Skolnick, 2025). These tools promise substantial benefits for medicine and biotechnology, but they also introduce dual-use risks that existing safeguards cannot address.

Most current approaches focus on model-level restrictions—keyword filtering, output screening, or access denials based on content (Wittmann et al., 2025; Feldman and Feldman, 2025b). While intuitive, these methods are poorly suited to biology. Keyword filtering can be circumvented (Feldman and Feldman, 2025b; Marsoof et al., 2023; Ball et al., 2025). Sequence homology detects known pathogens but misses novel designs by construction (Wittmann et al., 2025). Functional annotation remains incomplete, and reliable prediction of pathogenicity or toxicity is beyond current capabilities (Feldman and Feldman, 2025b; Wang et al., 2026; Liu, 2025). As biological design tools grow more powerful, these limitations create a difficult tradeoff: restrict access so heavily that legitimate research is impeded, or permit access so broadly that misuse becomes difficult to detect or attribute.

But there is another way. Instead of asking solely whether a particular output is dangerous—a question current systems cannot reliably answer (Feldman and Feldman, 2025b; Molotkov et al., 2024)—one can ask whether the user generating that output is one we trust. This shift from relying only on content inspection to supplementing it with verification reflects the technical limits of biological function prediction, and is not unique to our work. Rather, it is inspired by the analogous regulatory system created in the financial sector.

Indeed, Anti-Money Laundering (AML) frameworks from the financial sector provide a guide to how a similarly high-risk and complex problem was addressed, implementing the logic that we argue for here (Basel Committee on Banking Supervision, 2004; Board of Governors of the Federal Reserve System, 2026; FinCEN, 2016; FFIEC, 2026a; FINRA, 2026; Mansoor et al., 2023; Chen, 2020). AML systems aim to prevent the illicit flow of funds-for terrorism, organized crime, sanctions evasion, and the like-through layered identity verification of the users and behavioral monitoring of their actions (Basel Committee on Banking Supervision, 2004; FFIEC, 2026a; FATF, 2025). At the root of this regime are the Know Your Customer (KYC) requirements. They require that financial institutions verify customer identity and refuse service to sanctioned entities before providing access (Egan and Heim, 2023; FinCEN, 2016; Nibley, 2025). Transaction monitoring further detects suspicious patterns over time (IBM, 2026; FFIEC, 2026a; Basel Committee on Banking Supervision, 2004). The risk AML aims to prevent—enabling financial crime—is instructive for addressing the risk in biological AI: enabling pathogen design.

Although KYC-style approaches have long been discussed in AI governance and are already used within parts of the biotechnology supply chain—particularly in nucleic acid synthesis—they have not been formalized into an operational, biological AI-centric framework (Egan and Heim, 2023; Tarangelo et al., 2025; Facini, 2025; Moulange et al., 2023; Crawford et al., 2024). This paper addresses that gap.

The timing is urgent. Protein design capabilities have advanced rapidly over the past three years, with systems like AlphaFold, ESM3, and RFdiffusion enabling tasks that were recently out of reach (Ruffolo and Madani, 2024; Chen et al., 2023; Xiao et al., 2025; Watson et al., 2023; Huss et al., 2021; King et al., 2025). Many of these tools are now accessible through APIs, creating natural chokepoints for access control (Evolutionary Scale, 2026; Alphafold Server, 2026; Feldman and Feldman, 2025a). Scientists have called for stronger safeguards (Wang et al., 2025; Baker and Church, 2024), yet concrete, implementable frameworks remain scarce.

**Figure 1:**
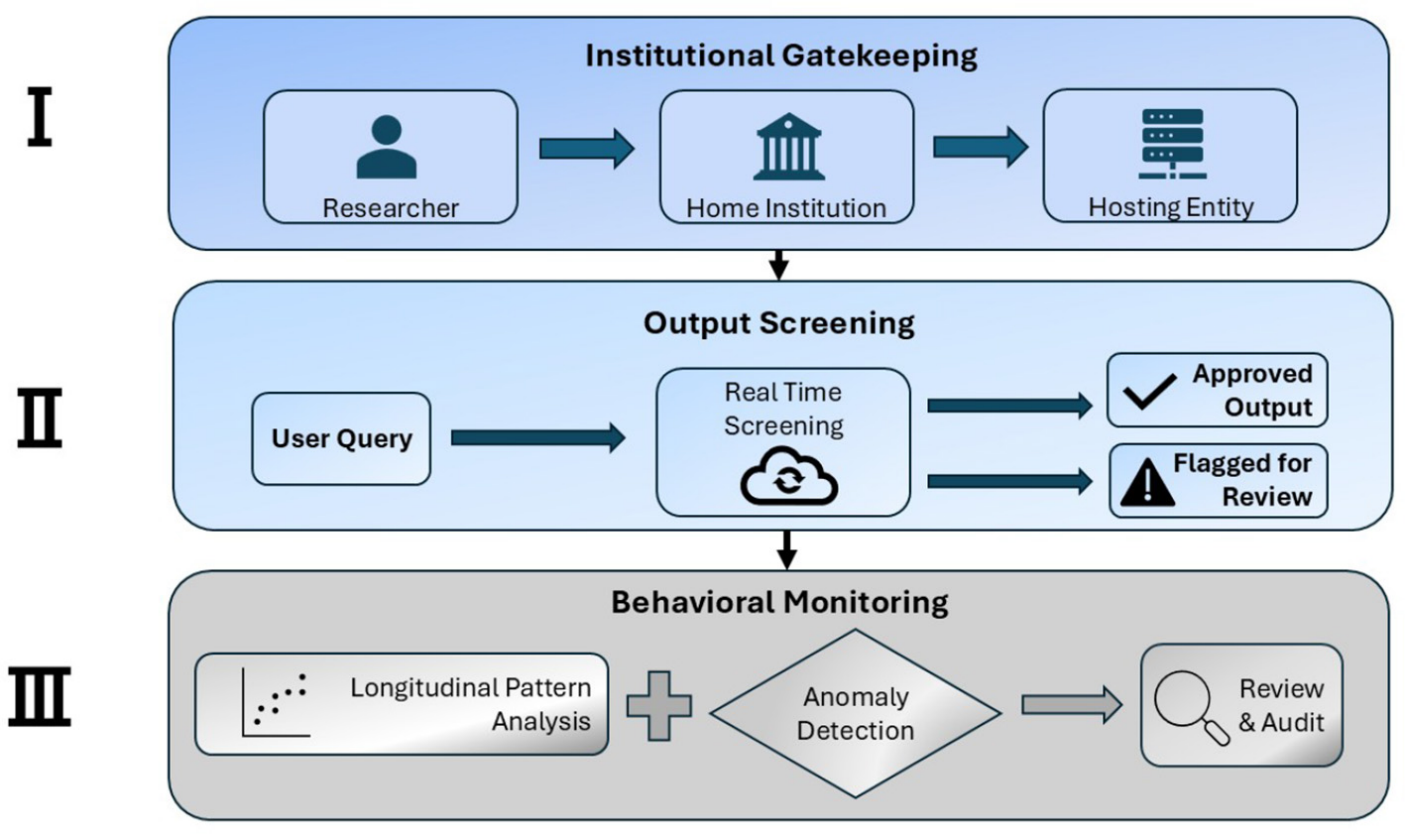
An architectural illustration of the three-tier KYC framework. Tier I (institutional gatekeeping): research institutions vouch for affiliated users and assume accountability for vetting. Tier II (output screening): real-time analysis of generated sequences using homology searches and functional annotation. Tier III (behavioral monitoring): longitudinal pattern analysis detects activity inconsistent with declared research purposes. Each tier provides independent security value while preserving access for legitimate researchers.

Hence, we propose a three-tier KYC framework (Figure 1) that leverages existing research institutions as trusted anchors, augments access control with output and behavioral monitoring, and establishes meaningful consequences for misuse or negligent oversight. This approach preserves access for legitimate researchers, raises the cost of misuse, and can be implemented immediately using existing institutional infrastructure—without waiting for new regulatory mandates.

## 2 Three-tier KYC framework

The framework adapts the layered structure of financial AML compliance to biological AI. In AML systems, KYC establishes verified identity at entry, while ongoing monitoring detects suspicious behavioral patterns (Basel Committee on Banking Supervision, 2004; FFIEC, 2026a; IBM, 2026). Neither mechanism alone provides adequate protection—identity verification without monitoring cannot detect gradual misuse; monitoring without verified identities cannot enable attribution. We implement the same layered approach: Tier I establishes verified researcher identity and institutional accountability. Tiers II-III monitor ongoing activity through output screening and behavioral pattern analysis.

The design leverages existing institutional infrastructure rather than introducing new layers of bureaucracy. Research institutions already vet researchers through hiring, training, and oversight processes, including biosafety and ethics review (Gao et al., 2025; Grady, 2015; Serpico et al., 2022; IBC, 2025). These mechanisms provide a natural basis for access control, allowing model hosts to rely on institutional judgment rather than attempting to infer legitimacy or intent from model interactions alone.

Minimizing friction for legitimate users is a core constraint. Most biological design tools require downstream wet-lab validation to have any practical impact, which in turn requires institutional affiliation or formal collaboration (Feldman and Feldman, 2025b; Wang et al., 2026; Suveena et al., 2025; King et al., 2025; Stark et al., 2025). Whether any given affiliated entity qualifies as a trusted institution under this framework is a separate question, governed by the hosting entity's calibrated trust criteria as described below. Aligning access with these existing structures preserves usability for legitimate research while raising the barrier to misuse.

Finally, the framework emphasizes accountability through consequences rather than brittle algorithmic restrictions. Automated filters with high false-positive rates are poorly suited to governing biological design. In contrast, clear attribution, traceability, and enforceable penalties create durable incentives for responsible use. These principles motivate a layered architecture in which each tier contributes independent security value while reinforcing the others.

This framework is contingent on the biological design tool to which it is applied being run in a managed access environment. Open-source models can be run or modified locally, bypassing any access control entirely. Going forward, we assume that any biological design tool is hosted by the entity that created it or a trusted third party.

### 2.1 Tier I: institutional gatekeeping

The first tier establishes trust at the point of access. Researchers seeking to use a biological AI tool apply through their home institution rather than directly to the model provider. The institution evaluates the request, confirms the researcher's identity and role, and vouches for the legitimacy of the proposed use. The hosting entity, in turn, maintains a list of trusted institutions whose endorsements it accepts and grants access based on institutional approval.

Throughout this framework, research institutions refer to organizations that conduct biological research under recognized oversight structures. This includes universities and colleges, government research laboratories, non-profit research institutes, hospital-affiliated research centers, and corporate R&D divisions. The common thread is institutional accountability: many of these organizations maintain biosafety committees, ethics review boards, or similar governance structures, and all employ researchers under formal relationships. These oversight mechanisms provide a foundation that can be leveraged for managing access to biological AI tools.

Startups and small biotechnology firms without equivalent administrative infrastructure present an added complexity. Such entities may partner with an established research institution, which would assume sponsorship responsibility under the same mechanism described below for visiting scholars and independent collaborators. Alternatively, a third-party vetting institution could emerge to vouch for smaller firms for a fee, defraying the cost of compliance overhead, assuming the same responsibilities as a research institution for the purposes of accountability.

This is similar to KYC's role in AML compliance. Just as banks verify customer identity before providing financial services and maintain exclusion lists identifying prohibited actors (Basel Committee on Banking Supervision, 2004; FinCEN, 2016; FFIEC, 2026a), biological AI hosts verify researcher identity through institutional vouching. The institution functions as the compliance officer: responsible for confirming the researcher is who they claim, their intended use is legitimate, and they do not appear on government-maintained exclusion lists (Office of Foreign Assets Control U.S. Department of the Treasury, 2026; U.S. Department of State, 2026).

This arrangement shifts the burden of vetting from model providers to institutions that are better positioned to assess researcher qualifications and intentions. It also creates a clear chain of accountability. Institutions that vouch for researchers implicitly accept responsibility for their vetting, and misuse by affiliated users carries reputational and access consequences.

AML frameworks rely on objective exclusion criteria rather than subjective trustworthiness judgments. Regulators maintain lists of sanctioned individuals, designated terrorist entities, and convicted financial criminals (Office of Foreign Assets Control U.S. Department of the Treasury, 2026; U.S. Department of State, 2026; United Nations Security Council, 2026). Biological AI governance adopts the same approach. Hosting entities should deny access to individuals or organizations appearing on government-maintained exclusion lists, including OFAC's Specially Designated Nationals, the State Department's Foreign Terrorist Organizations list, and the Commerce Department's Entity List, as well as individuals convicted under biological weapons statutes (Office of Foreign Assets Control U.S. Department of the Treasury, 2026; U.S. Department of State, 2026; Bureau of Industry U.S. Department of Commerce and Security, 2026). Beyond these bright-line exclusions, institutions retain discretion for context-specific vetting—analogous to enhanced due diligence banks conduct for higher-risk customers (FATF, 2025; Basel Committee on Banking Supervision, 2004)—given the varied nature of potential use cases and the complexity of assessing them.

#### 2.1.1 Outlining inclusion criteria

Criteria for institutional trust are determined by the hosting entity and scale with the assessed risk of the model or tool to which access is being granted. Each host evaluates prospective institutions according to its own trust framework, which may include factors such as the presence of an Institutional Biosafety Committee (IBC) (IBC, 2025; Egan and Heim, 2023), receipt of federally funded research subject to established oversight regimes, prior compliance history, or an existing relationship with the host. Crucially, these institutional signals are interpreted in light of the model's risk profile, rather than in isolation.

Ongoing work on biological AI risk assessment increasingly enables models to be classified by their potential for misuse or harm, allowing hosts to calibrate trust requirements accordingly (De Haro, 2024; Ackerman et al., 2025; Rand Europe, 2024). For lower-risk systems—such as small protein language models comparable to those in the ESM family (Hayes et al., 2024)—the threshold for institutional trust may reasonably be lower. In contrast, access to more advanced systems, such as models capable of de novo genome design (Hwang et al., 2024; Brixi et al., 2025), may warrant substantially stricter requirements, effectively limiting access to institutions with demonstrated oversight capacity and clearly legitimate use cases.

To accommodate common research practices within this risk-sensitive framework, institutions may sponsor non-affiliated individuals, including visiting scholars, collaborators, or independent researchers. In such cases, the sponsoring institution submits the access request, vets both the individual and the proposed use, and assumes responsibility for ongoing oversight. Any misuse would affect the sponsor's standing with hosting entities, reinforcing accountability while preserving flexibility. Importantly, the willingness—or refusal—of institutions to sponsor a given individual provides an additional signal of perceived risk, further integrating model risk, institutional trust, and host-specific requirements into a unified access control framework.

### 2.2 Tier II: output screening

The second tier introduces continuous monitoring of model outputs to detect potentially concerning activity. Generated sequences or designs are analyzed in real time using available screening tools. Current methods include sequence homology searches, such as BLAST, and ontology-based functional annotation to identify similarity to known pathogens or hazardous functions (Wittmann et al., 2025; Feldman and Feldman, 2025b; Zhao et al., 2025; Miller and Fuchs, 1997). These tools are imperfect, but they provide useful signals when interpreted in context.

As predictive technologies advance, this tier can incorporate more sophisticated tools, including protein function predictors and toxicity assessments (Feldman and Feldman, 2025b,a; Wang et al., 2023). The framework is intentionally modular, allowing screening capabilities to evolve without altering the underlying access-control structure. In addition, emerging watermarking and output-provenance technologies for generative models can be integrated into these pipelines, providing an extra layer of accountability—particularly when generated sequences are synthesized or otherwise deployed (Chen et al., 2025; Zhang et al., 2024).

When outputs are flagged as potentially pathogenic, viral, or otherwise concerning, and the user's declared use case does not justify access to such outputs, an inquiry can be initiated. Flagged outputs may be temporarily frozen and held for review, and the associated user activity is logged. Crucially, the focus is not on automated enforcement but on generating signals that prompt human oversight. Users are aware that their activity is monitored and attributable, reinforcing accountability (D'Arcy et al., 2009).

For example, a researcher developing vaccine candidates against influenza may legitimately generate sequences resembling viral proteins. The system would flag these outputs due to their similarity to pathogen components, but a review would confirm the researcher's declared use case of vaccine development, allowing access to continue. In contrast, if a user whose stated purpose is analyzing mitochondrial genomes produces similar sequences, the discrepancy between their actions and declared use case would trigger an escalated review.

This mirrors AML transaction monitoring, which generates alerts and escalates potentially suspicious activity for review rather than relying on fully automated enforcement. Monitoring systems are designed to identify patterns or anomalies that warrant investigation and contextual assessment, and flagged activity may proceed if review determines it to be consistent with legitimate purpose (FFIEC, 2026a; FATF, 2025). The objective is not perfect automated detection—which AML frameworks explicitly recognize as unattainable—but the generation of signals that enable human oversight, accountability, and attribution (Basel Committee on Banking Supervision, 2004; FFIEC, 2026a). Similarly, pathogen-homologous outputs may trigger review without necessarily resulting in access denial when a legitimate research use, such as vaccine development, is documented (Feldman and Feldman, 2025b).

### 2.3 Tier III: behavioral pattern monitoring

The third tier addresses risks that emerge over time rather than from individual outputs. User activity is analyzed longitudinally to identify patterns that deviate from declared use cases or expected research behavior. Signals may include repeated interactions with flagged outputs, aggregate risk signals over time, unexpected access patterns, or other inconsistencies between stated research goals and observed usage. This approach is analogous to AML behavioral monitoring (National Institute of Standards and Technology, 2025; FFIEC, 2021; IBM, 2026; FFIEC, 2026a).

Rather than relying on single events, this tier uses threshold-based accumulation of signals to trigger review. Importantly, decisions to suspend or revoke access are not automated—human review is required before enforcement actions are taken, allowing contextual judgment and reducing the risk of erroneous penalties. Determining whether outputs are inconsistent with a declared use case may require considerable domain expertise and institutional familiarity with the researcher's work, and is not a trivial undertaking. Where possible, the home institution and hosting entity should work in tandem to assess legitimacy, particularly after repeated flagging, as institutions are better positioned to evaluate whether a researcher's activity is consistent with their broader research program.

Together, these three tiers form a deep defense framework. Institutional gatekeeping establishes trust at entry, output screening provides immediate response, and behavioral monitoring captures longer-term risk. Each tier compensates for the limitations of the others, creating a system that is both practical and robust as biological AI capabilities continue to advance.

### 2.4 Shared responsibility as the foundation of the framework

Effective governance of biological AI systems requires a genuinely shared allocation of responsibility between research institutions and hosting entities. Research institutions serve as the first line of defense by vetting applicants, evaluating proposed use cases, and ensuring that researchers operate within appropriate oversight structures. This institutional gatekeeping is essential, but it is inherently limited by the institution's visibility into downstream model use and therefore cannot function as a complete safeguard on its own.

Hosting entities bear an independent and complementary responsibility that research institutions cannot fulfill alone. Hosts must assess which institutions they trust, calibrate access requirements to the risk profile of the model, and implement technical oversight mechanisms such as output screening, activity logging, and behavioral monitoring. Because hosts have direct, system-level visibility into model interactions, they are uniquely positioned to detect anomalous usage patterns or emergent risks that may not be observable at the institutional level.

The framework is effective precisely because responsibility is distributed rather than centralized. Neither research institutions nor hosts are individually capable of managing the full risk surface of advanced biological AI systems. Instead, institutions evaluate researchers and intent, while hosts validate institutions and monitor interaction with the technology. This shared and interdependent oversight structure minimizes failure, reinforces accountability on both sides, and ensures that risk management remains an ongoing, collective obligation.

## 3 Adoption incentives

### 3.1 Researcher acceptance

Preventing biosecurity risk currently requires some access friction—it is impossible to deter misuse without restrictions (Anderljung and Hazell, 2023). The question is whether friction remains tolerable for legitimate users. Several factors support researcher acceptance of the framework proposed here.

First, friction is minimal. Researchers already navigate institutional approvals for laboratory work, grants, and protocols (Gao et al., 2025; Hoffmann et al., 2023; Tang et al., 2024). A one-time institutional access request represents marginal additional burden. Most researchers continue working without interruption after initial approval.

Second, restrictions target those who should not have unrestricted access. Unlike popular LLMs which are general-purpose and can be used by a wide swath of the population for legitimate uses, there is little reason for individuals who are not doing cutting-edge biological research and have no institutional affiliation to independently access tools capable of designing pandemic-potential agents (Wang et al., 2025, 2026; Feldman and Feldman, 2025)—just as they should not work unsupervised with live biological agents (IBC, 2025; Gao et al., 2025).

Third, the AML analogy is instructive. Banks verify every customer's identity and subject customer transactions to ongoing monitoring, introducing friction for account holders worldwide (FinCEN, 2016; FFIEC, 2026a; Basel Committee on Banking Supervision, 2004). Despite these requirements, modern payment systems operate at global scale, processing large volumes of transactions each day (Federal Reserve Board, 2025, 2024). Risk-based AML and KYC controls are designed to be proportionate so they do not unnecessarily disrupt legitimate activity, while monitoring and suspicious activity reporting increase the detection, traceability, and disruption of illicit finance (FATF, 2026).

### 3.2 Hosting entity incentives

For hosting entities, this framework is far more tenable than the speculative alternative: building systems that reliably predict whether designed proteins are dangerous (Feldman and Feldman, 2025b; Wang et al., 2026). Current biological AI cannot determine sequence pathogenicity reliably (Feldman and Feldman, 2025b; Cao et al., 2025). Solving this requires breakthroughs in structural biology, immunology, and machine learning—an expensive, uncertain, potentially impossible task (Wittmann et al., 2025; De Haro, 2024; Gao et al., 2025).

KYC resolves this by deferring vetting and attribution to institutions, reducing the risk exposure for hosting entities. The institution vouches for a user, the user operates under institutional oversight, and misuse traces to both. The hosting entity verifies institutional credentials and maintains access records.

Reputational incentives also favor adoption. High-profile misuse of a host's tool could trigger regulatory backlash and institutional distrust. Proactive safeguards function as reputational insurance (Bravo-Urquiza and Moreno-Ureba, 2021; Chukwudi, 2025).

## 4 Enforcement mechanisms and regulatory pathways

### 4.1 Voluntary adoption with distributed enforcement

The framework is designed for immediate voluntary adoption by hosting entities without requiring regulatory mandates. Companies providing API access to biological design tools—including AlphaFold Server, ESM APIs, RFdiffusion implementations, and similar platforms—can implement institutional gatekeeping, output screening, and behavioral monitoring unilaterally through terms of service (Evolutionary Scale, 2026; Alphafold Server, 2026; Zhang et al., 2025; Javadi et al., 2020). This voluntary approach enables rapid deployment while regulatory pathways develop.

The primary enforcement mechanism is access revocation. Biological AI tools represent powerful capabilities that accelerate research and enable designs previously requiring specialized laboratory infrastructure (Ruffolo and Madani, 2024; Watson et al., 2023; Zhang et al., 2025). Access to these tools is a privilege, not a right. Users who violate terms of service through misrepresentation, circumvention of safety measures, or generation of outputs inconsistent with declared research purposes lose access. Institutions that repeatedly vouch for problematic users or fail to address misconduct by affiliated researchers risk institution-wide access revocation.

Critically, enforcement gains strength through information sharing between hosting platforms. Just as financial institutions share information about suspicious actors through regulatory reporting systems and authorized inter-institution information-sharing mechanisms (FFIEC, 2026b; Financial Crimes Enforcement Network, 2026; LLOYDS Banking Group, 2025), biological AI hosts can establish shared exclusion lists. When one platform revokes access due to misuse, that individual or institution appears on a shared registry accessible to other platforms. In practice, such information-sharing would likely rely on predefined, objective reporting categories. These may include documented violations of terms of service, confirmed misrepresentation, or other clearly specified compliance failures. Limiting shared information to such standardized criteria reduces reliance on subjective judgments about institutional trustworthiness and mitigates legal and governance concerns.

This distributed enforcement structure addresses a key challenge: individual platforms may hesitate to revoke access due to competitive pressure or uncertainty about violations. If problematic users can immediately migrate to alternative platforms, enforcement loses deterrent value. Shared exclusion lists eliminate this escape route, making violations consequential. An institution that loses trusted status with major platforms effectively loses access to the biological AI ecosystem, creating strong incentives for rigorous internal oversight.

### 4.2 Existing models of delegated oversight

U.S. biosecurity and research security governance has often relied on institutional intermediaries rather than comprehensive, individualized federal vetting of all researchers. In several high-risk domains, the federal government establishes baseline rules while conditioning participation in the regulated activity on institutions assuming responsibility for day-to-day access control and oversight (U.S. Government Publishing Office, 2025; Federal Select Agent Program, 2024). This division of labor reflects practical constraints: federal agencies set guardrails and enforce compliance, but institutions translate those requirements into operational decisions about who may access sensitive capabilities and under what conditions.

The Federal Select Agent Program exemplifies this hybrid model (CDC, 2024a; Select Agents and Toxins, 2024). Federal law determines which biological agents are regulated and identifies individuals who are legally barred from access (Select Agents and Toxins, 2024; CDC, 2024a). At the same time, research entities must register, designate responsible officials, and maintain internal controls governing access to regulated materials (Select Agents and Toxins, 2024; CDC, 2024a). Individual access decisions occur within the institution, informed by—but not supplanted by—federal security risk assessments conducted through the Department of Justice (Federal Select Agent Program, 2024; CDC, 2024a; Select Agents and Toxins, 2024). The government thus retains authority over exclusion criteria, security vetting, and enforcement, while institutions bear primary responsibility for monitoring, local oversight, and controlling access in practice (Select Agents and Toxins, 2024; CDC, 2024a,b).

A similar structure appears in export control and research security regimes. This typically involves screening potential partners and visitors against government-maintained restricted lists, assessing whether planned activities implicate controlled items or technology, and establishing internal procedures or safeguards to manage compliance risks (Massachusetts Institute of Technology Office of the Vice President for Research, 2025). Federal agencies define what is controlled and when a license is required, but institutions handle day-to-day compliance and can be penalized for violations. Across export controls and research security, this split—federal rules and enforcement paired with institutional gatekeeping—has become a common way to manage sensitive research in complex settings.

### 4.3 Federal pathways for standardization

While voluntary adoption enables immediate implementation, federal action could accelerate adoption and establish consistent standards across providers. Several pathways exist under current executive authority.

Federal research funding agencies already impose biosafety and dual-use research oversight requirements on recipient institutions (Gao et al., 2025; U.S. Government, 2024; National Institutes of Health, 2025). Agencies could extend these requirements to include verification that researchers accessing biological AI tools operate under institutional oversight frameworks meeting minimum standards. The NIH, NSF, and DOE, who are major funders of U.S. biological research, could condition awards on institutional implementation of KYC-style access controls for affiliated researchers (National Archives, 2026; Levin, 2025). This would likely not require new legislation and could be achieved through administrative rulemaking specifying that institutions receiving federal funds must establish processes for vetting researcher access to biological design tools and maintaining accountability for their use.

The National Institute of Standards and Technology (NIST), through CAISI, maintains authority to develop voluntary consensus standards for AI systems (NIST, 2017, 2016). NIST could convene stakeholders—hosting entities, research institutions, biosecurity experts—to develop technical standards for implementing the three-tier framework. These standards could be voluntary but would likely become de facto industry requirements through market pressure and federal procurement specifications. They may also become binding rules through agency rulemaking.

Notably, the federal government has already moved to make biosecurity a priority, focusing on nucleic acid synthesis screening. The Biden Administration's 2024 Screening Framework Guidance for Providers of Synthetic Nucleic Acids establishes requirements for DNA synthesis companies to screen orders against databases of concerning sequences and verify customer identity (Office of Science and The White House Technology Policy, 2024; Department of Health et al., 2023). This framework recognizes that biological capability risks require verification of who has access, not just what sequences are synthesized. Our proposed KYC framework represents the logical upstream extension: if we screen synthesized DNA orders, we should also govern access to the AI tools that design those sequences in the first place.

The Trump administration's AI policy discussions have similarly emphasized synthesis screening as a national security priority, with proposals to expand screening requirements and enhance enforcement mechanisms (House, 2025). Extending governance upstream to biological AI design tools aligns with this trajectory. AI-generated designs must eventually be synthesized to pose physical risks (Feldman and Feldman, 2025b; Mouneyrac et al., 2017; Suveena et al., 2025), but governing only at the synthesis stage creates gaps—researchers can accumulate dangerous knowledge, share designs through alternative channels, or synthesize abroad. Comprehensive biosecurity requires governance at both the design and synthesis stages (Feldman and Feldman, 2025b; Wittmann et al., 2025; Baker and Church, 2024).

Federal action could also address the open-source model problem. As noted earlier, this framework is contingent on biological design tools remaining under centralized hosting; open-source models bypass access control entirely. Agencies could condition federal funding or establish export controls for the most capable biological design models, ensuring they remain under hosted access with implemented safeguards. This would not prevent all capability diffusion but would slow proliferation of the highest-risk tools (Feldman and Feldman, 2025a).

### 4.4 Federal oversight for high-risk biological AI

As biological AI tools increase in capability, the risks they pose may reach a level that necessitates direct federal oversight. Implementing this pathway is likely the most challenging option, requiring dedicated funding, infrastructure, and sustained administrative capacity. In such cases, the federal government could assume a role in vetting individual researchers, either independently or in coordination with research institutions and hosting entities. This responsibility could be managed by an existing agency or a dedicated subsidiary, such as within the Department of Energy or the Centers for Disease Control.

Under this model, hosting institutions would continue to monitor usage and track user activity, while the federal entity would oversee researcher vetting. The government could maintain a registry of high-risk models and establish statutory, funding-related, or collaborative obligations—such as for federal contractors—to ensure compliance with vetting procedures.

Recognizing practical limits on resources, federal vetting could focus on researchers seeking access to the riskiest models, as defined by formal risk assessments. Importantly, vetting would remain a shared responsibility: research institutions could continue to serve as primary evaluators, with the federal government providing secondary review and oversight. While this approach is more difficult to implement than host-level or collaborative oversight, it provides the most rigorous multi-layered safeguards for the highest-risk models, enabling scalable and accountable governance.

## 5 Limitations and open questions

The framework has several important limitations, some of which have been noted above. Open-source models bypass access control entirely, rendering KYC ineffective without centralized hosting (Feldman and Feldman, 2025a; Black et al., 2025). To apply this framework, biological AI tools must not be made open source.

KYC is also not a complete defense against determined, well-resourced state actors that can establish front institutions. Its primary strength lies in deterring lone actors and raising the cost of misuse through accountability and traceability, rather than eliminating all possible threats. Still, the instructive AML example from finance shows that there are ways to detect even well-resourced actors that hide behind complex ownership structures.

Calibration remains an open challenge. Thresholds for output flagging and behavioral monitoring require empirical tuning and human oversight to balance false positives against security sensitivity. These issues are best addressed through iterative refinement rather than static rules. More broadly, we intentionally leave many operational details—such as review timelines, appeals processes, and specific criteria for institutional trust—to individual implementors. Different hosting entities face different threat models, resource constraints, and user populations, making overly prescriptive specifications counterproductive. The framework provides architectural principles while preserving flexibility for context-specific adaptation.

Lastly, a malicious actor with genuine institutional affiliation could use biological AI tools for legitimate purposes while gradually probing system boundaries or accumulating knowledge applicable to harmful ends. Tier III monitoring provides some protection against this pattern, as careful misuse by affiliated users, including insiders who would seek to deliberately subvert the system, carries reputational and access consequences. No access control system, however, fully solves the insider threat problem.

## 6 Conclusion

Biological AI tools have reached a capability threshold that demands governance infrastructure, yet existing content-based restrictions remain fundamentally inadequate for biology. Inspired by the financial sector's AML system, this paper has proposed a three-tier KYC framework that addresses this mismatch by supplementing content-based output screening—a necessary but insufficient safeguard—with user identity verification and institutional accountability.

The framework's core insight adapts a proven model from financial compliance: layered defenses combining entry-point verification, continuous monitoring, and behavioral pattern analysis. Tier I leverages research institutions as trust anchors, creating accountability through institutional vouching. Tier II applies available output screening tools while acknowledging their limitations. Tier III detects concerning patterns over time through longitudinal behavioral analysis. Each tier provides independent security value while compensating for the others' weaknesses.

Critically, this approach can be implemented immediately. Hosting entities can adopt institutional gatekeeping unilaterally through terms of service without waiting for regulatory mandates. The framework builds on existing institutional infrastructure rather than adding new regulatory burden. For legitimate researchers, friction should be minimal, and for potential bad actors, the framework raises costs through attribution, monitoring, and enforceable consequences.

Important limitations remain. Open-source models bypass access control entirely; well-resourced state actors could establish front institutions; and the calibration of screening thresholds and behavioral patterns will require empirical tuning to balance false positives against security sensitivity.

Despite these limitations, the framework offers substantial security gains over the status quo. The alternative is either restricting access so heavily that legitimate research suffers, or maintaining open access with minimal safeguards as capabilities grow more dangerous. This framework charts a middle path: preserving access for accountable researchers while raising barriers to misuse through institutional responsibility, continuous monitoring, and enforceable consequences.

As biological AI capabilities continue advancing, governance infrastructure will become increasingly critical. The framework proposed here provides a concrete starting point—implementable today, scalable as capabilities evolve, and grounded in the practical realities of both biological research and risk management. The question is not whether such infrastructure will eventually be necessary, but whether we implement it proactively or reactively. We advocate for the former.

## Data Availability

The original contributions presented in the study are included in the article/supplementary material, further inquiries can be directed to the corresponding author.
